# Impact of virtual problem-based learning of cardiopulmonary resuscitation on fourth-year nursing students’ satisfaction and performance: a quasi-experimental study

**DOI:** 10.1186/s12909-024-05375-5

**Published:** 2024-04-19

**Authors:** Seyedeh Nayereh Falahan, Edris Habibi, Naser Kamyari, Vahid Yousofvand

**Affiliations:** 1 School of Nursing and Midwifery, Hamadan University of Medical Sciences, Hamadan, Iran; 2 Student Research Committee, Hamadan University of Medical Sciences, Hamadan, Iran; 3 Department of Biostatistics and Epidemiology, School of Health, Abadan University of Medical Sciences, Abadan, Iran; 4 Student Research Committee, School of Nursing and Midwifery, Shahid Beheshti University of Medical Sciences, Tehran, Iran

**Keywords:** Problem-based learning, Cardiopulmonary resuscitation, Nursing students, Performance, Satisfaction

## Abstract

**Background:**

Regarding competency of nursing students in cardiopulmonary resuscitation (CPR), nursing students frequently exhibit inadequate performance and low satisfaction levels regarding CPR training methods. The problem-based learning (PBL) method, characterized by a constructivist approach, has been underutilized for CPR training, particularly in a virtual format. Hence, this study aims to assess the influence of virtual problem-based learning in cardiopulmonary resuscitation on the satisfaction and performance of fourth-year nursing students.

**Methods:**

This quasi-experimental study, conducted in 2022, involved 80 final-year nursing students from Hamadan University of Medical Sciences, Iran. The participants were randomly assigned to either the experimental group (*N* = 40) or the control group (*N* = 40). The experimental group was further divided into six smaller groups on WhatsApp. Both groups initially received routine training sessions, after which the experimental group engaged in four problem-based learning sessions across three different scenarios. Data collection included demographic information, a teaching satisfaction questionnaire, and cardiopulmonary resuscitation checklists administered immediately and one month after the intervention.

**Results:**

The study was initiated and concluded with 80 participants. The study commenced with no significant disparity in the mean scores of cardiopulmonary resuscitation performance, encompassing chest compressions (*P* = 0.451) and airway management (*P* = 0.378), as well as teaching satisfaction (*p* = 0.115) among the nursing students between the experimental and control groups. However, subsequent to the intervention, both immediately and one month later, the experimental group displayed notable enhancements in mean scores for cardiopulmonary resuscitation performance, comprising chest compressions (*p* < 0.001) and airway management (*p* < 0.001), as well as teaching satisfaction (*p* < 0.001) compared to the control group.

**Conclusion:**

Based on the study’s findings, it is recommended that nursing educators implement this approach in their teaching practices.

## Background

Cardiopulmonary arrest, being a life-threatening condition, demands prompt interventions from healthcare professionals, particularly nurses, to safeguard lives and avert irreversible harm to vital organs [1]. Consequently, nurses and nursing students must possess the competence to initiate and execute effective cardiopulmonary resuscitation (CPR) at the onset of their nursing careers [2]. Despite this imperative, various studies have documented suboptimal CPR performance [3–5]. Liou et al. additionally noted that nursing students frequently encounter challenges and difficulties in comprehending the educational content and mastering the clinical skills associated with cardiopulmonary resuscitation [6].

Spinelli et al.‘s investigation revealed inadequacies not only among nursing students but also among nursing staff and medical personnel concerning CPR training and knowledge, indicating a notable gap in this domain [7]. Furthermore, Nasr-Esfahani et al.‘s study underscored suboptimal performance levels among nursing students in correctly executing cardiopulmonary resuscitation [8]. Another study evaluating the knowledge and proficiency of nursing students in adhering to standard cardiopulmonary resuscitation protocols reported exceptionally low levels of competence [9]. Some research attributes nursing students’ deficient competence and subpar performance in accurately conducting cardiopulmonary resuscitation to insufficient training and the dissatisfaction of nursing students with the teaching methodology [10, 11].

A study conducted in Iran indicated that students’ express dissatisfaction with the traditional, teacher-centered approach to CPR education [12]. Similarly, another study found that graduate nurses exhibited lower satisfaction levels with conventional cardiopulmonary resuscitation instruction compared to an electronic delivery method [13]. It was suggested in a study that the dissatisfaction among medical or nursing students in CPR training groups might be attributed to the high number of participants, highlighting the potential benefits of smaller group sizes [14]. In a qualitative study, nursing students emphasized the importance of smaller groups, particularly during the COVID-19 pandemic, to allow teachers to allocate more time for individualized instruction with each student [15]. Thus, for the effective achievement of CPR objectives aimed at saving lives, it is advisable to educate nursing students in smaller groups, enabling instructors to correct mistakes and provide personalized feedback on performance [16].

In the study conducted by Nabecker et al., it was suggested that reducing the size of training groups, particularly during the initial sessions of basic life support, may enhance trainers’ proficiency in detecting performance errors [17]. Furthermore, a qualitative inquiry by Dziurka et al. revealed that a significant proportion of nursing students expressed discontent with the pedagogical approach of solely imparting theoretical knowledge, particularly its teacher-centered nature. They tend to have opportunities to utilize various instructional methods to enhance nursing skills and performance throughout their academic trajectory [15].

Numerous studies indicate that over time, the knowledge and performance of nursing students, as well as nursing graduates, tend to decline [18]. To uphold patient safety and enhance students’ self-confidence in executing clinical procedures such as CPR, it is imperative for university professors to deliver accurate training in clinical skills through innovative teaching methods [19]. Despite the critical importance of CPR training, insufficient attention has been directed toward employing engaging and suitable instructional techniques. Consequently, the current imperative extends beyond merely expanding cardiopulmonary resuscitation training; rather, it underscores the paramount need to enhance the quality of this training [20].

It is crucial to acknowledge that while all forms of training contribute to learning, the depth and sustainability of learning may vary across different training methods [21]. Various studies have scrutinized educational approaches aimed at enhancing the quality of CPR training. These approaches encompass both direct and indirect methods, including workshops, lectures, videos, brochures, pamphlets, e-learning, and multimedia software [9].

Presently, lectures represent a prevalent method for CPR training in Iran; however, achieving comprehensive and effective training to enhance the quality of instruction necessitates the incorporation of additional complementary methods [22]. Even brief training sessions conducted in clinical skill centers fall short of providing a comprehensive grasp of all pertinent skills. There remains a dearth of knowledge remains regarding the optimal teaching methods for imparting this critical and essential skill [23].

On the other hand, certain studies argue that acquiring and applying CPR-related training for nursing students necessitates engaging in a problem-solving process through critical thinking [6]. Despite nursing curricula placing emphasis on fostering critical thinking skills in nursing students and graduates, findings from previous studies in Iran indicate varying levels of competence in these skills, ranging from weak to moderate, among nursing students and nurses [24]. Nevertheless, a study conducted in Malaysia reported a high level of critical thinking among nursing students [25]. Ahmady and colleagues attributed the consequences of poor critical thinking skills and the inability of nursing students to solve problems to a widening gap between theory and practice, a deficiency in timely decision-making, and suboptimal clinical reasoning [26].

Thus, nursing students are encouraged to employ problem-solving and critical thinking strategies to unravel intricate solutions to complex issues such as CPR [27]. In this context, considerable attention has been directed towards problem-based learning (PBL), anticipating its role in enhancing students’ critical thinking skills for competent CPR performance and increased satisfaction in learning it [28]. Problem-based learning is an educational approach that shifts the teacher’s role to a more student-centered dynamic, grounded in self-directed learning principles [29].

According to Ghani et al., PBL emphasizes learning behaviors conducive to critical thinking, problem-solving, communication, and collaborative skills in student preparation. The study by Ghani et al. identified internal empowerment, delegation, and performance skills as three pivotal elements effective in attaining learning outcomes through PBL [30]. A comprehensive review indicated that PBL is an effective and gratifying method in medical education, suggesting that medical students, through PBL, acquire not only knowledge but also essential competencies requisite for the medical profession [29]. Prior research has demonstrated that students instructed through PBL exhibit superior problem-solving abilities compared to those receiving traditional lectures [31].

Perdana et al.‘s study found the problem-solving method to be more effective in enhancing students’ digital literacy than the online laboratory simulation method with concept maps [32]. Another study concluded that PBL more effectively promotes nursing students’ progress and academic motivation compared to the lecture method [33]. However, a singular study posited that the PBL approach in first-year medical students did not significantly enhance critical thinking/knowledge, problem-solving, and self-direction compared to conventional teaching methods [34].

Simultaneously, the surge in communication facilities and equipment has propelled the popularity of online learning and virtual technologies in education [35]. Leveraging modern technologies, including virtual space, is imperative in the practical training of nursing students. This utilization aims to equip students with the skills to navigate challenging and novel situations, foster decision-making proficiency, and enhance problem-solving capabilities [15]. A study conducted in Indonesia demonstrated that the implementation of the problem-based learning model through electronic learning media resulted in improved learning outcomes, coupled with high satisfaction levels among students [36]. In a study by Aslan et al., participants engaged in live online classes using the PBL approach exhibited elevated levels of learning achievement, problem-solving skills, and direct interaction during online sessions. Nevertheless, no significant difference was observed in the communication skills of the groups [37].

In Iran, several studies have revealed that a majority of nursing educators predominantly employ content-based educational methods and exhibit a preference for a formal educational setting characterized by limited student participation [38, 39]. Given that Iranian nursing students have demonstrated subpar and moderate performance levels in prior investigations, coupled with their low satisfaction with CPR training methods, and considering that PBL, a constructivist approach, has been underutilized for CPR training, the current study seeks to assess the impact of a virtual problem-based learning (PBL) approach of cardiopulmonary resuscitation on the satisfaction and performance of fourth-year nursing students.

## Methods

### Design and setting

This pre-post-test quasi-experimental study was undertaken at the School of Nursing and Midwifery, Hamadan University of Medical Sciences, between February 2021 and June 2022. The study sample comprised 80 fourth-year nursing students enrolled in semesters seven and eight.

### Participants and sampling

The sample size for the current study was determined in accordance with a precedent study [40], considering a confidence level of 95%, a test power of 80%, an effect size of 0.69, and factoring in a potential attrition rate of 18%. As a result, a total of 80 participants, with 40 individuals allocated to each group, were deemed sufficient for the study.

**Fig. 1:**
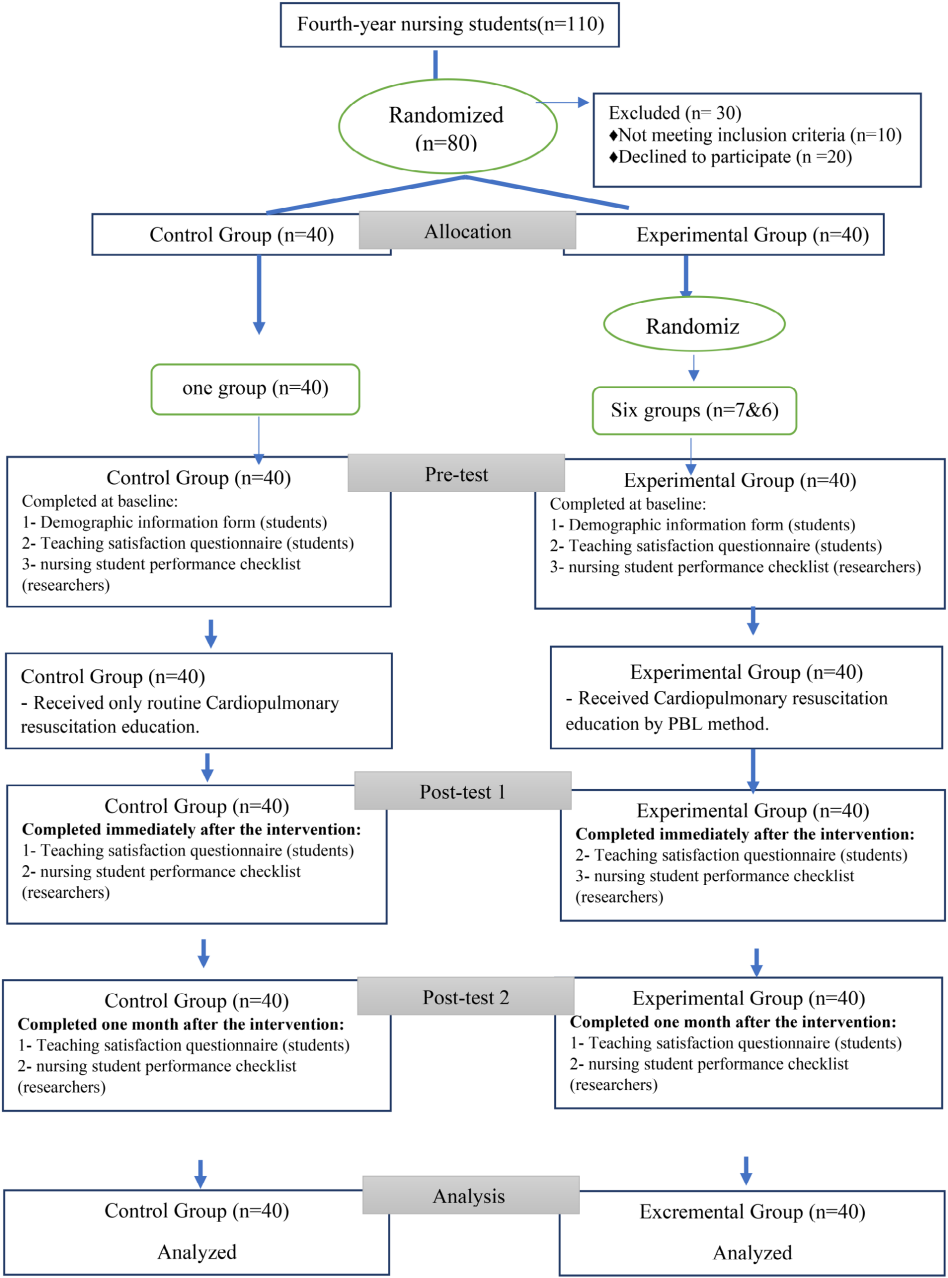
CONSORT diagram

Following the inclusion criteria, 80 fourth-year students were selected for the study and then randomly assigned to either the experimental group (*N* = 40) or the control group (*N* = 40). Both groups were evenly distributed with a mix of 7th and 8th semester nursing students (fourth year) (Fig. 1). The experimental group was further divided into six subgroups using a random number table in WhatsApp. Similarly, the control group was also organized into a WhatsApp group. It is important to note that all study participants had received routine CPR training during their sixth academic semester.

The inclusion criteria for this study required participants to have successfully completed the cardiopulmonary resuscitation course as outlined in the academic curriculum, and not to have taken additional CPR courses from external organizations outside of the nursing and midwifery faculty. The exclusion criteria encompassed being absent for more than one training session and failing to complete the questionnaire. Prior to participation in the study, written informed consent was obtained from the students, with a total of 80 nursing students ultimately taking part in the research (comprising fifty 8th-semester students and sixty 7th-semester students).

### Tools and measurements

#### Demographic information form

The demographic information form included age, gender, marital status, student employment history, time spent in the student workplace, student workplace department, and cardiopulmonary resuscitation experience. In terms of face validity, ten Hamadan Nursing and Midwifery Faculty members reviewed and approved this form.

#### Teaching satisfaction for nursing student’s questionnaire

The questionnaire utilized in this study was developed by Borim Nejad et al. (2015) through an extensive literature review, examination of other satisfaction questionnaires, and consultations with academic experts. Comprising 16 items on a 3-point Likert scale (completely = 3, somewhat = 2, not at all = 1), the questionnaire assesses aspects such as increased interest in the subject and the application of gained knowledge and skills in communication. Scores on this questionnaire range from 16 to 48, with a higher score indicating greater satisfaction. Student satisfaction levels with teaching are categorized into three tiers: low (scores between 0 and 16), medium (16–32), and high (scores above 32). The validity of the questionnaire was established through input from 15 specialists, resulting in necessary adjustments for form and content. The content validity index (CVI), calculated based on Waltz and Basel standards, was determined to be 0.85. Reliability was assessed using the test-retest method, with a reported correlation of 0.90 [41]. In the current study, the internal consistency of the questionnaire was found to be 0.93, indicating a high level of reliability.

In this study, the final-year nursing students had previously received cardiopulmonary resuscitation (CPR) training in the emergency department during the preceding semester as part of their curriculum, utilizing a teacher-centered approach. Their satisfaction with this teaching method was assessed before the intervention. Following the implementation of the intervention, their satisfaction with teaching, particularly in comparison to the Problem-Based Learning (PBL) method, was evaluated. Some of the questions pertaining to teaching satisfaction in the questionnaire are provided below:

1. I found this teaching method enjoyable.
2. This method adequately addressed my information needs.
3. The knowledge and skills acquired through this method are applicable to my profession.
4. I believe my performance improved with this method.
5. Learning occurred more efficiently with this method.
6. I perceive learning to be more effective with this approach.
7. This method enhanced my motivation to learn.
8. I found the subject matter more engaging with this approach.
9. This method contributed to the enhancement of my clinical judgment.
10. Overall, I am satisfied with this teaching method.
11. I would recommend implementing this method in other educational settings.

#### Cardiopulmonary resuscitation procedure checklist

The Ministry of Health of Iran has presented a standardized 20-item scale for evaluating the competency of nursing students in cardiopulmonary resuscitation. This scale comprises ten inquiries pertaining to cardiac massage techniques and ten inquiries concerning airway management during adult resuscitation. Each item is evaluated using a three-point Likert scale, with options for weak = 1, moderate = 2, and good = 3. The cumulative score for each section is determined by adding together the individual item scores. The performance of nursing students in each section is classified into three tiers: inadequate (scores between 0 and 10), moderate (11–20), and proficient (21–30).

Before and after the intervention, the performance of students in cardiopulmonary resuscitation (CPR), covering chest compressions and airway management, was evaluated by two assessors. The assessment panel comprised an instructor with a medical-surgical nursing degree, 17 years of experience in the emergency department, and a PhD student in medical-surgical nursing with five years of experience in emergency and intensive care units. Evaluations were conducted both concurrently and separately. In cases where both assessors reached a consensus on the assigned grades for the students, the same grade was recorded. If a discrepancy arose between the two assessors, the opinion of a third assessor, a PhD holder in emergency nursing with 25 years of experience, was solicited. Subsequently, all three assessors reached a consensus, and grades were allocated to the students accordingly. The evaluation took place in the Faculty of Nursing and Midwifery simulation lab, utilizing pre-prepared mannequins.

### Intervention

Prior to the intervention, both the experimental and control groups completed a demographic information form and the Satisfaction teaching for nursing students’ questionnaire. Additionally, an observational cardiopulmonary resuscitation practical test was conducted as a pre-test to complete the cardiopulmonary resuscitation checklist.

Subsequently, as an initial session, both the control and experimental groups received identical educational content via WhatsApp, including voice slides, educational videos, and questions. Twenty participants from each group were selected based on random numbers and were questioned to ensure their comprehension of the training content. Those who required further understanding of the educational content were provided with additional instruction after one or two days and were subsequently questioned about it.

The intervention consisted of four sessions, each lasting one hour per week. The first intervention session was uniform for both groups. However, from the second session onwards, the control group did not receive any further training. In contrast, the remaining three sessions for the experimental group focused on problem-based learning. During these sessions, students utilized PDF files to implement the ESI triage manual, 4th edition, to three scenarios [42]. At each session, researchers presented a scenario along with necessary explanations. Subsequently, students asked questions to clarify any ambiguities and received appropriate answers. Following receipt of the scenario, students were required to study it carefully during the week and solve it based on the steps described in Table 1.

**Table 1:** Steps designed based on problem-based learning

Educational steps	Activity title	Activity description
**First**	Clarification of ambiguous concepts	Each group explains the vague and unknown concepts of the problem to itself.
**Second**	Define the problem	Each group seeks appropriate information, regardless of irrelevant details, and expresses the problem in its own language.
**Third**	This step is the rain of thoughts or brain	Each group records its initial responses without evaluating them.
**Fourth**	Problem analysis	Each group tries to divide the problem into manageable parts.
**Fifth**	Division of responsibility	Each member of a group is responsible for answering the problem and finding a solution.
**Sixth**	Individual study and finding Answer for the problem	Each person uses different sources to find answers. Then the people of each group come together and by reviewing the collected answers and comparing them with the initial answers presented in the third step, they summarize the answers and prepare a summary of them for presentation in the group in the WhatsApp software environment.
**Seventh**	Providing solutions and giving reasons	Each group shares its findings.

In the initial session, a hypothetical scenario was presented involving a 65-year-old male patient diagnosed with asystole, with a medical history including myocardial infarction and diabetes. The students were tasked with mentally placing themselves in the clinical setting and initiating the initial steps of cardiopulmonary resuscitation (CPR) while providing the rationale for their actions. Subsequently, they were required to continue CPR and provide ongoing care during and after resuscitation efforts. Initially, one of the researchers provided a comprehensive case overview.

Following this, students were instructed to review the latest Cardiopulmonary Resuscitation Guidelines (2020) provided by the researchers in group settings, and then apply the steps outlined in the problem-based learning (PBL) approach. The researchers monitored each group’s progress, offering guidance when necessary, and facilitated group discussions to analyze the scenario comprehensively. Prior training had been provided to the students on how to effectively analyze CPR-related problems, considering various dimensions such as their responsibilities in the situation, necessary actions, potential risks to the patient, composition of the resuscitation team, required resources, and possible further interventions.

Students were encouraged to identify and discuss any uncertainties or ambiguities within the scenario, providing justification and rationale for their perspectives. In subsequent stages, students were prompted to share both correct and incorrect responses within their groups without judgment. All steps outlined in Table 1 were sequentially implemented. At the conclusion of each session, students and researchers collectively reviewed the PBL process, and each group presented their final conclusions. The resources available to students included CPR guidelines for different scenarios, PDF files containing the Emergency Severity Index (ESI) triage manual (4th edition), and guidance from the researchers. Subsequent sessions aimed to increase the level of challenge by presenting more complex scenarios to the students.

### Data collection methods

At the outset (pre-test), immediately following the intervention (post-test 1), and one month thereafter (post-test 2), the nursing students were tasked with completing a demographic information form and a teaching satisfaction questionnaire. Furthermore, the researchers administered a cardiopulmonary resuscitation performance checklist in a practical setting. Both the experimental and control groups were requested to complete the teaching satisfaction questionnaire immediately and one month after the intervention at the designated location within the nursing school. Following the practical examination of cardiopulmonary resuscitation, the researchers conducted an assessment of the students’ performance in this course. Adhering to research ethics, the material presented to the experimental group was also made available to the control group upon conclusion of the study.

### Data analysis

Upon completion of data collection, statistical analysis was conducted using SPSS version 22. Descriptive statistics, including mean, standard deviation, frequency, and percentage, were used to describe the data. The normality of the data was assessed using the Kolmogorov-Smirnov and Shapiro-Wilk tests. Subsequently, independent t-tests, chi-square tests, Fisher’s exact tests, repeated measures ANOVA, and Bonferroni’s post hoc tests were employed for data analysis. The significance level for this study was set at 0.05.

### Ethical considerations

The current study received approval from the Ethics Committee of Hamadan University of Medical Sciences (ethical code: IR.UMSHA.REC.1400.884). Prior to commencing the research, the researchers provided a detailed explanation of the study objectives to the participants. Subsequently, written informed consent was obtained from the students in order for them to participate in the study and for the results to be published. Furthermore, the researchers assured the participants that all information provided would be kept confidential and emphasized their right to withdraw from the study at any point.

## Results

The baseline analysis of socio-demographic characteristics revealed that there were no significant differences between the experimental and control groups, indicating homogeneity (Table 2).

**Table 2:** Socio-demographic characteristics of participants at baseline (*N* = 80)

Variables		Groups		*P*-value
		Experimental (N = 40) N (%)	Control (N = 40) N (%)	
**Age (year)**	Less than 25	33 (82.5%)	34 (85%)	0.762^*^
	More than 25	7 (17.5%)	6 (15%)	
**Gender**	Male	23 (57.5%)	21 (52.5%)	0.653^*^
	Female	17 (42.5%)	19 (47.5%)	
**Marital status**	Single	34 (85%)	38 (95%)	0.263^**^
	Married	6 (15%)	2 (5%)	
**Student work experience**	Yes	8 (20%)	12 (30%)	0.302^*^
	No	32 (80%)	28 (70%)	
**Student work duration (month)**	1–3	6 (75%)	8 (66.7%)	1.000^**^
	More than 3	2 (25%)	4 (33.3%)	
**Student work ward**	Internal	6 (75%)	9 (75%)	1.000^**^
	Surgery	2 (25%)	4 (25%)	
**CPR** ^**#**^ **experience**	Yes	10 (25%)	8 (20%)	0.592^*^
	No	30 (75%)	32 (80%)	

^*^: *P*-value derived from chi-square test

^**^: *P*-value derived from Fisher’s Exact Test

^#^: CPR: Cardiopulmonary resuscitation

Prior to the intervention, the performance of fourth-year nursing students in chest compression and airway management was found to be moderate to weak, as indicated by mean scores of 11.93 (SD = 1.11) and 10.95 (SD = 1.10) in the experimental group, and 11.73 (SD = 1.24) and 10.75 (SD = 0.89) in the control group, respectively. There were no significant differences between the groups at baseline (*P* = 0.451 for chest compression and *P* = 0.378 for airway management). However, immediately and one month after the intervention, significant differences were observed between the groups (*P* < 0.001). Additionally, the experimental group showed a significant improvement in performance scores from baseline to one month after the intervention (*p* < 0.001), while the control group did not show significant improvement (*P* > 0.05) (Table 3).

**Table 3:** Mean comparison of student performance scores before, immediately after, and one month after the intervention (*N* = 80)

Student performance		Groups		*P*-value
		Experimental (N = 40)	Control (N = 40)	
**Chest compressions (Mean ± SD)**	Before the intervention	11.93 ± 1.11	11.73 ± 1.24	0.451^*^
	immediately after the intervention	25.85 ± 2.42^1^	11.83 ± 1.21	< 0.001^*^
	one month after the intervention	25.35 ± 2.27^1^	12.03 ± 1.65	< 0.001^*^
	***P-value***	**< 0.001** ^†^ F = 900.53	0.107^†^ F = 2.52	
		**< 0.001** ^¶^ F = 75.95		
**Air way management (Mean ± SD)**	Before the intervention	10.95 ± 1.10	10.75 ± 0.89	0.378^*^
	immediately after the intervention	26.10 ± 1.58^1^	10.93 ± 0.91	< 0.001^*^
	one month after the intervention	25.35 ± 1.57^1^	11.38 ± 1.03^1^	< 0.001^*^
	***P-value***	**< 0.001** ^†^ F = 1258.60	176^†^ F = 8.31	
		**< 0.001** ^¶^ F = 81.16		

^*^: *P*-value derived from independent sample t-test

^†^: *P*-value derived from single repeated measurement

^¶^: *P*-value derived from overall repeated measurement. ^1^: significantly different with first time point (before intervention)

Teaching satisfaction among fourth-year nursing students was moderate before the intervention, with mean scores of 19.83 (SD = 4.29) in the experimental group and 18.63 (SD = 2.07) in the control group. There was no significant difference between the groups before the intervention (*p* = 0.115). However, significant differences were observed between the groups immediately and one month after the intervention (*p* < 0.001). The mean teaching satisfaction scores of nursing students in the experimental group significantly improved from baseline to one month after the intervention (*p* < 0.001), while no significant differences were observed for the control group (*p* = 0.175) (Table 4).

**Table 4:** Mean comparison of teaching satisfaction scores before, immediately after, and one month after the intervention (*N* = 88)

Teaching satisfaction		Groups		*P*-value
		Intervention (n = 40)	Control (n = 40)	
**Teaching satisfaction** **(Mean ± SD)**	Before the intervention	19.83 ± 4.29	18.63 ± 2.07	0.115^*^
	Immediately after the intervention	43.63 ± 3.77^1^	18.87 ± 2.00	**< 0.001** ^*^
	One month after the intervention	41.32 ± 3.04^1,2^	18.95 ± 2.23^1^	**< 0.001** ^*^
	***P-value***	**< 0.001** ^†^ F = 521.20	0.175^†^ F = 1.83	
		**< 0.001** ^¶^ F = 71.50		

^*^: *P*-value derived from independent sample t-test

†: *P*-value derived from single repeated measurement

¶: *P*-value derived from overall repeated measurement

^1^: significantly different with first time point (before intervention)

^2^: Significantly different with second time point (immediately after)

## Discussion

The present study assessed the impact of problem-based learning virtual training on cardiopulmonary resuscitation on the teaching satisfaction and performance of fourth-year nursing students. Prior to the intervention, there were no significant differences in the mean scores of chest compression and airway management in cardiopulmonary resuscitation between the experimental and control groups, indicating that students had a moderate to weak skill level in this area. This may be attributed to the limited education provided before graduation, as well as the need for access to recognized instructional resources from international organizations and institutes that evaluate these principles.

However, this study’s results demonstrated a significant difference between groups immediately after the intervention and one month afterward. Additionally, the experimental group’s mean performance scores in chest compression and airway management increased from baseline to one month after the intervention. Therefore, problem-based learning virtual education courses on cardiopulmonary resuscitation effectively improve nursing students’ performance.

Aligned with our study, Habibli et al. demonstrated that nursing students exhibited significantly higher performance scores immediately after the intervention and at a three-month follow-up compared to the control group, indicating that simulation-based training enhanced the proficiency of nursing students in Basic Life Support-Cardiopulmonary Resuscitation (BLS-CPR) [43]. Similarly, Ren et al.‘s investigation corroborated our findings by illustrating that Problem-Based Learning (PBL) modules yielded more effective medical education outcomes across various medical science specialties, fostering both theoretical knowledge and practical skills compared to traditional lecture-based approaches. Participants receiving PBL reported more positive feedback and satisfaction than those exposed to conventional methods [44].

Echoing our research, Hazrati et al.‘s comprehensive review highlighted the efficacy of PBL in Iranian medical education, showcasing its role in enhancing student performance, fostering critical thinking, and garnering student satisfaction [45]. Additionally, Towfik et al. demonstrated the significant impact of problem-based learning on strengthening critical thinking skills, clinical satisfaction, academic progress, and course learning achievements (skills and values) among nursing students [46]. GU Deng-yu et al.‘s study further supported the benefits of problem-based learning, particularly in conjunction with case-based learning, for improving performance and clinical practice skills among nurse anesthesia trainees compared to lecture-based learning [47].

Park et al.‘s investigation aimed to assess the educational effects of a blended e-learning program for nursing graduate students on self-efficacy, problem-solving, and psychomotor skills for core nursing skills. Their results indicated that participants who underwent combined e-learning experienced enhancements in problem-solving abilities and self-efficacy related to nursing performance, particularly in cardiopulmonary resuscitation and defibrillation. Although most psychomotor skills demonstrated excellent post-intervention performance rates ranging from 80 to 90%, certain aspects such as chest compression location, compression rate per minute, artificial respiration, and patient outcome verification still exhibited lower performance levels [48].

Baccin et al. found that PBL education through mobile phones has been shown to enhance nursing students’ knowledge of diagnosing and taking timely action at the patient’s bedside. The authors also emphasized that using PBL through mobile phones could serve as a valuable learning strategy for nurses, and suggested that nursing students should be actively involved in this process [49]. Several other studies have highlighted that nursing students can access clinical opportunities and educational topics via mobile phones, with many nurses and nursing students reporting a positive and progressive impact on their knowledge acquisition process [33, 36, 37, 50].

Additionally, Lee et al. demonstrated that simulation problem-based learning can enhance nursing students’ communication attitudes, suggesting its potential for application in clinical practice to improve communication attitudes and facilitate the application of learned knowledge to simulated nursing situations through experiential learning [51]. The utilization of mobile phone capabilities and communication applications has the potential to enhance nursing education beyond traditional settings such as the university and the patient’s bedside [52, 53]. Furthermore, problem-based learning (PBL) methods have been shown to improve students’ abilities in analyzing information, critical thinking, and problem-solving skills [30].

The findings of the current study contrast with those of Kang et al.‘s study, “The Effect of Virtual Education on Nursing Students’ Learning in Caring for Children with Asthma,” which found that virtual education did not significantly improve students’ knowledge. It appears that simply preparing and sending educational materials may not be sufficient to enhance the knowledge of the research sample. The current study suggests that incorporating online simulated or face-to-face courses, as well as the opportunity for live participation and discussion, leads to greater participant satisfaction due to the convenience and unrestricted access to content [54]. Additionally, Manuaba et al.‘s study demonstrated that problem-based learning (PBL) is not effective at enhancing critical thinking, problem-solving, and self-direction among first-year medical students [34]. Meanwhile, Mendel et al.‘s research at the Washburn School of Nursing in the USA concluded that virtual education has a minimal impact on reducing moral distress in neonatal intensive care unit nurses, whereas face-to-face meetings related to palliative care for infants reduced moral distress among nurses in neonatal intensive care units. The author suggests that palliative care meetings in the final stages of life may alleviate some of these distresses [55]. These studies collectively indicate that a primary obstacle to raising awareness of virtual training courses is inadequate follow-up and participants’ need for more focus on the content of the training sessions.

Prior to the intervention, fourth-year nursing students expressed a moderate level of satisfaction with the teaching of cardiopulmonary resuscitation. Following the intervention, the mean scores of teaching satisfaction among fourth-year students in the experimental group showed a significant increase from baseline to one-month post-intervention in comparison to the control group. Thus, the findings of the study suggest that virtual education utilizing problem-based learning (PBL) for cardiopulmonary resuscitation courses had a positive impact on students’ satisfaction with the teaching.

In alignment with our research, Jannah et al.‘s findings revealed that approximately two-thirds of nursing students expressed satisfaction with the Problem-Based Learning (PBL) approach and its instructional methodology [56]. Similarly, Forouzan Jahromi et al. demonstrated that nursing students reported significantly higher levels of learning satisfaction when engaged in problem-based learning than outcome-based learning, particularly within the intensive care unit setting [57]. Tadesse et al.‘s study further supported these results by indicating higher academic satisfaction among students in problem-based learning programs than lecture-based learning environments [58]. Additionally, González Hernando et al. observed a positive reception to PBL, with 78% of students expressing a preference for this method post-implementation. Following active engagement in PBL, students demonstrated heightened motivation and satisfaction across educational content, instructional methods, the learning process, instructor involvement, and student roles [59].

Moreover, Son’s study underscored the efficacy of integrating simulator programs with PBL as a strategy to enhance clinical reasoning abilities and foster learning satisfaction among nursing students [60]. Consistent with our investigation, a comprehensive review by Hazrati et al. highlighted the advantageous impact of PBL on increasing teaching satisfaction in medical education within Iran [45]. Furthermore, GU Deng-yu et al.‘s research indicated that students in the intervention group, exposed to a combination of problem-based learning and case-based learning, exhibited higher overall satisfaction, greater acceptance of the teaching model, and superior knowledge mastery compared to those in the control group receiving lecture-based instruction [47].

In their study, Berger et al. found that nursing students exhibited higher levels of satisfaction with teaching when utilizing mobile phones and PBL methods compared to traditional teaching approaches. They recommended the use of mobile phones and PBL to enhance student satisfaction [61]. Similarly, Trullàs et al. reported that the PBL method resulted in high levels of teaching satisfaction among nursing and medical students [29]. Furthermore, Xing et al. demonstrated that CPBL and SBAR improved nursing students’ problem-solving and critical thinking abilities, with both students and teachers expressing satisfaction with the new teaching method [62]. Sangestani et al. also observed increased satisfaction among midwifery students when utilizing the PBL method [63]. Lastly, Sharma’s research indicated that PBL increased nursing students’ self-efficacy and improved learning outcomes [64].

However, Jannah et al.‘s study highlights that despite nursing students’ satisfaction with the teaching method, several challenges persist within the learning process. For instance, students express concerns regarding inadequate time to fully comprehend the subject matter. Additionally, when learning topics involve practical field-related facts, students may exhibit reluctance to engage in discussions. Moreover, there are limitations in learning resources, indicating a need for effective management of e-learning tools and regulation of students’ access to ensure the availability of pertinent learning materials for sharing among peers [56].

The findings of this study underscore the potential of modern, learner-centered teaching methods, exemplified by Problem-Based Learning (PBL), to supplant traditional teacher-centered approaches in nursing education. PBL emerges as a transformative pedagogical strategy that not only enhances nurses’ preparedness for skills and behavioral challenges but also empowers students to attain diverse educational objectives through the integration of mobile technology and social media. Consequently, it is recommended that future research endeavors capitalize on the implications of employing PBL within the educational landscape, contributing to the advancement of educational methodologies. Furthermore, it is advisable to conduct additional research to comprehensively elucidate the impact of the PBL teaching method on nursing students’ teaching satisfaction and performance.

### Limitations

In the present study, there is a potential limitation regarding the participants’ comprehensive understanding of the training content. To address this limitation, a random selection of 20 students from both the experimental and control groups was made, and they were assessed through a series of questions related to the educational content. Subsequently, the training content was reviewed with those who did not fully comprehend it, and after a few days, they were re-evaluated through a question-answer session. Another limitation of this study pertains to the need for follow-up on nursing students’ performance and satisfaction post-intervention. Therefore, it is recommended that future research includes observations of nursing students at the patient’s bedside during cardiopulmonary resuscitation to strengthen this innovative learning method further. Furthermore, additional research is necessary to fully understand the impact of the PBL education method on nursing student performance.

## Conclusion

The present study’s findings suggest that PBL virtual training can effectively maintain CPR skills and enhance teaching satisfaction among nursing students. As such, it is recommended that nursing educators consider incorporating this educational approach to enhance knowledge and satisfaction levels among their students.

## Data Availability

The datasets generated and/or analyzed during the current study are not publicly available due to keeping participants’ information confidential but are available from the corresponding author at reasonable request.
